# Association between echocardiographic parameters and biomarkers in probands with atrial fibrillation and different PR interval lengths: Insight from the epidemiologic LIFE Adult Study

**DOI:** 10.1371/journal.pone.0212627

**Published:** 2019-02-28

**Authors:** Jelena Kornej, Samira Zeynalova, Joachim Thiery, Ralph Burkhardt, Ronny Baber, Christoph Engel, Andreas Hagendorff, Markus Loeffler, Daniela Husser

**Affiliations:** 1 Department of Electrophysiology, Heart Center, Leipzig, Germany; 2 Institute for Medical Informatics, Statistics, and Epidemiology, University of Leipzig, Leipzig, Germany; 3 LIFE – Leipzig Research Center of Civilization Diseases, University of Leipzig, Leipzig, Germany; 4 Institute of Laboratory Medicine, Clinical Chemistry and Molecular Diagnostics, University of Leipzig, Leipzig, Germany; 5 Department of Cardiology, University of Leipzig, Leipzig, Germany; Universita degli Studi di Roma La Sapienza, ITALY

## Abstract

**Background:**

PR interval prolongation is associated with increased risk for atrial fibrillation (AF). Different biomarkers are used to predict AF incidence and its outcomes. The aim of this study was to investigate the association between echocardiographic parameters and blood biomarkers in PR interval groups and AF.

**Methods:**

The LIFE-Adult-Study is a population-based cohort study of randomly selected participants from Leipzig, Germany. In this cross-sectional analysis, individuals ≥40 years with available echocardiographic (LA diameter, EF) and laboratory data (creatinine, Troponin, NT-proBNP) were included.

**Results:**

The study population comprised 1.429 individuals (median age 56 (IQR 48–66) years, 40% males) with complete ECG, echocardiographic and laboratory data. There were 48 (3.4%) individuals with AF, 177 (12.4%) with short, 138 (9.7%) with prolonged and 1.066 (74.5%) with normal PR interval. Individuals with PR interval prolongation had larger LA diameter, higher Troponin and NT-proBNP levels than individuals with normal PR interval, but lower than AF group (p<0.001). In contrast, eGFR was significantly higher in the group with PR interval prolongation than in AF, but lower than in individuals with normal PR interval (p<0.001). In the multivariate analysis, PR interval prolongation and AF shared similar characteristics, the only parameter different between both groups was NT-proBNP.

**Conclusions:**

Individuals with PR interval prolongation and AF showed similarities in echocardiographic parameters, renal function and blood biomarker levels. Longitudinal studies are necessary to prove whether the PR interval prolongation may be considered as preliminary stage for AF.

## Introduction

The PR interval is the delay between the excitation of the atria and ventricles and is determined by the sum of atrial and atrioventricular nodal conduction [1]. So far, PR prolongation without structural heart disease or additional conduction disturbances has been considered as a benign occurrence [2]. However, recent studies have demonstrated an association between PR prolongation and the incidence of atrial fibrillation (AF) [2,3].

AF is the most common cardiac arrhythmia in clinical routine. It is associated with an increased risk of dementia, heart failure, and thromboembolism, leading to an increased hospitalization, higher treatment costs and mortality [4]. AF leads to electrical and structural remodeling of the atrial myocardium (inflammation, fibrosis, atrial dilatation). These processes may be analyzed using histology and peripheral blood biomarkers. Prediction of subclinical AF using simple tools, as ECG, clinical parameters (e.g. renal function) and biomarkers (e.g. inflammation, cardiac damage and stress), echocardiography, might be used for identification of high-risk patients to avoid disease progression and initiate individualized arrhythmia prevention.

Peripheral biomarkers play an important role in experimental, clinical and epidemiologic settings. Multiple studies have analyzed associations between natriuretic peptides, pro-inflammatory, pro-thrombotic and biomarkers of endothelial dysfunction/damage with cardiovascular disease and adverse outcomes [5–7]. Although there is a huge interest in identifying biomarkers relevant for AF prediction, it is still unknown whether electrocardiographic PR disturbances and peripheral biomarkers could be helpful to identify individuals at risk for AF development. Recently, we demonstrated that Troponin T is associated with PR interval prolongation suggesting subclinical heart disease in an epidemiologic setting [8]. The aim of current analysis was to investigate further the association between echocardiographic parameters, renal function and blood markers of cardiac stress, myocardial damage and inflammation in individuals with normal PR, PR interval prolongation and AF.

## Methods

All data generated or analyzed during this study are included in this published article. The study was approved by the responsible institutional ethics board of the Medical Faculty of the University of Leipzig. All methods were performed in accordance with the relevant guidelines and regulations.

### Study population

The study design comprised an age and gender stratified random sample of residents of the City of Leipzig, in the age group of 20 to 79 years as previously described [9]. The main objective of the LIFE Adult Study is to investigate prevalence, early onset markers, genetic predispositions as well as the role of lifestyle factors of major civilization diseases, especially metabolic and vascular diseases, heart function, cognitive impairment, depression, and allergies. Written informed consent was obtained from all individuals who were interested to participate in the LIFE project.

Individuals younger than 40 years, with previous myocardial or stroke, pacemaker stimulation or not available laboratory data, as well as individuals with atrio-ventricular conduction decelerating medication (e.g. beta blockers, calcium-antagonists, antiarrhythmic drugs) were excluded. Finally, 1.429 individuals were included into the analyses.

### ECG

To investigate cardiac arrhythmias a 10-second 12-lead electrocardiogram (ECG) was recorded using the PageWriter TC50 ECG system (Philips Medical Systems DMC GmbH, Hamburg, Germany) after a supine resting period of at least 10 min. The ECG was evaluated manually in all probands based on published criteria with particular focus on rhythm and conduction disturbances, ST-segment and J-point changes, T and U waves, PR and QT interval, hypertrophy, and QRS morphology [10].

### Echocardiography

Echocardiography was one of the routine examinations in the LIFE-Adult-Study [9]. Cardiac ultrasound examination was performed using the GE Vivid 7 dimensions BTO8 echocardiography station (GE Healthcare). Echocardiography was conducted by one of 3 study nurses, who were extensively trained for two months by a supervisor-sonographer with European certification. Standardized reading of the echocardiographic assessments was performed according to ASE recommendations and the European Society of Cardiology by means of the software EchoPAC Version 113 (GE Healthcare).

### Laboratory measurements

Blood was drawn from all study participants after >8 hours fasting and analyzed on the same day. All samples were processed in a highly standardized manner–details are described elsewhere [9]. Laboratory measurements of creatinine, Troponin T and NT-proBNP serum concentrations were performed on the same day at the Institute of Laboratory Medicine, University Hospital Leipzig (accredited by ISO 15189 and 17025) according to the Quality Standards for Medical Laboratories of the German Chamber of Physicians (RiLiBÄK) using assays from Roche Diagnostics on Cobas 6000 or 8000 (Roche Diagnostics) clinical chemistry analyzers. eGFR was estimated using the CKD-EPI (Chronic Kidney Disease Epidemiology Collaboration) equation: **eGFR = 141 X min(Scr/ĸ, 1)**^**α**^ **X max(Scr/ĸ, 1)**^**-1.209**^ **X 0.993Age X 1.018 [if female] X 1.159 [if black]**, where Scr is serum creatinine, ĸ is 0.7 for females and 0.9 for males, α is -0.329 for females and -0.411 for males, min indicates the minimum of Scr/ĸ or 1, and max indicates the maximum of Scr/ĸ or 1 [11].

### Definitions and cut-offs

PR interval was analyzed only in individuals with sinus rhythm and defined as PR prolongation if ≥200 ms, while short PR interval was <120 ms. AF was diagnosed during ECG analysis by irregular cycle length and/or presence of f-waves. In logistic regression analysis the continuous biomarker levels were dichotomized as follows: LA diameter ≥40 mm, NT-proBNP >125 pg/ml and eGFR <60 ml/min/1.73m^2^. There were 2 cut-offs for Troponin T: >4 pg/ml and ≥10 pg/ml (the latter was chosen as more clinically important).

### Statistical analysis

Baseline characteristics were described for the study population and stratified by the length of PR and by the presence or absence of AF using medians and interquartile ranges for continuous variables as well as absolute and relative frequencies for categorical variables. Comparisons of continuous variables were made using non-parametric tests (Mann-Whitney-U-tests or Kruskal-Wallis). Unordered categorical variables were compared using the Pearson χ2 test.

To analyze the independent association of factors we used a multivariate logistic regression analysis and developed three models: 1) participants with normal PR interval vs. PR prolongation; 2) normal PR interval vs. AF; and 3) AF vs. PR interval prolongation. We used a stepwise approach for including single factors accordingly to the modelling approach of D. Collett adapted to logistic regression [12]. This approach assumes that all variables are on an equal footing, and there is no *a priori* reason to include any specific variables. For this analysis, we included different clinical and demographical characteristics as well as biomarkers. The Likelihood ratio test was used for all variable inclusion/exclusion decisions. A two-tailed p-value <0.05 was considered as statistically significant. Additionally, the Bonferroni correction was used to avoid an increased risk of a type I error when making multiple statistical tests in 3 models (p<0.05 / 3 = 0.017). All statistical analyses were performed with IBM SPSS Statistics for Windows Version 23 (IBM Corp, Armonk, NY, USA).

## Results

The baseline characteristics of study population are presented in Table 1. The study population comprised 1.420 individuals (median age 55 years (IQR 48–66), 40.1% males) with complete ECG, echocardiographic and laboratory data. There were 48 individuals (3.4%) with AF, 177 (12.4%) with short PR interval (sPR), 138 (9.7%) individuals with PR prolongation (pPR) and 1.057 (74.5%) with normal PR interval (nPR).

**Table 1:** Baseline characteristics of study population accordingly to PR interval length or AF.

	sPR (<120ms) n = 177	nPR interval* n = 1.057	pPR (>200ms)* n = 138	AF n = 48	*p*-value	*p*-value*
Age, years	53 (47–63)	54 (47–63)	67 (58–72)	70 (67–75)	<0.001	<0.001
Gender, m/f (%)	26 / 74	41 / 59	68 / 32	60 / 40	<0.001	<0.001
BMI, kg/m^2^	25 (23–28), 25±4	26 (24–28), 26±4	27 (25–30), 27±4	28 (27–31), 29±3	<0.001	0.002
BMI≥30 kg/m^2^, %	50	60	69	85	<0.001	<0.001
Hypertension, %	40	43	64	79	<0.001	<0.001
Diabetes mellitus, %	3	5	9	29	<0.001	0.113
Heart rate	67 (61–74), 68±10	63 (58–70), 64±10	62 (55–70), 63±12	83 (69–90), 82±19	<0.001	0.126
Heart rate >100 bpm, %	1.7	0.2	0	13	<0.001	0.494
Creatinine, μmol/l	74 (64–80)	76 (68–85)	83 (71–95)	91 (79–102)	<0.001	<0.001
eGFR, ml/min/1.73m^2^	85 (70–100)	82 (70–97)	75 (61–90)	61 (51–75)	<0.001	<0.001
LA diameter, mm	34 (32–38)	37 (34–40)	39 (35–42)	46 (42–51)	<0.001	<0.001
Enddiastolic LA volume, ml/m^2^	21 (18–26)	24 (19–30)	29 (22–32)	77 (67–95)	<0.001	<0.001
LV-EF, %	62 (58–65)	63 (59–67)	62 (57–67)	59 (54–62)	<0.001	0.052
LVIDd, mm	49 (45–53)	52 (48–55)	51 (48–55)	53 (46–58)	<0.001	0.411
Troponin T, pg/ml	3.8 (3.0–5.3)	3.9 (3.0–5.8)	7.0 (4.6–10.5)	10.0 (6.3–17.2)	<0.001	<0.001
NT-proBNP, pg/ml	56 (35–106)	58 (34–105)	77 (45–126)	951 (640–1679)	<0.001	0.003
PR interval, ms	114 (109–117)	152 (140–167)	216 (205–222)		<0.001	<0.001

**Abbreviations**: BMI—body mass index, LAD—left atrial diameter, LV-EF—left ventricular ejection fraction, LVIDd—left ventricular end-diastolic diameter, eGFR—estimated glomerular filtration rate. Data presented as mean (IQR)

*p*-value*—comparison between 2 groups with nPR and pPR interval

### Univariate analysis

#### Left atrial diameter (LAD)

**Fig 1:**
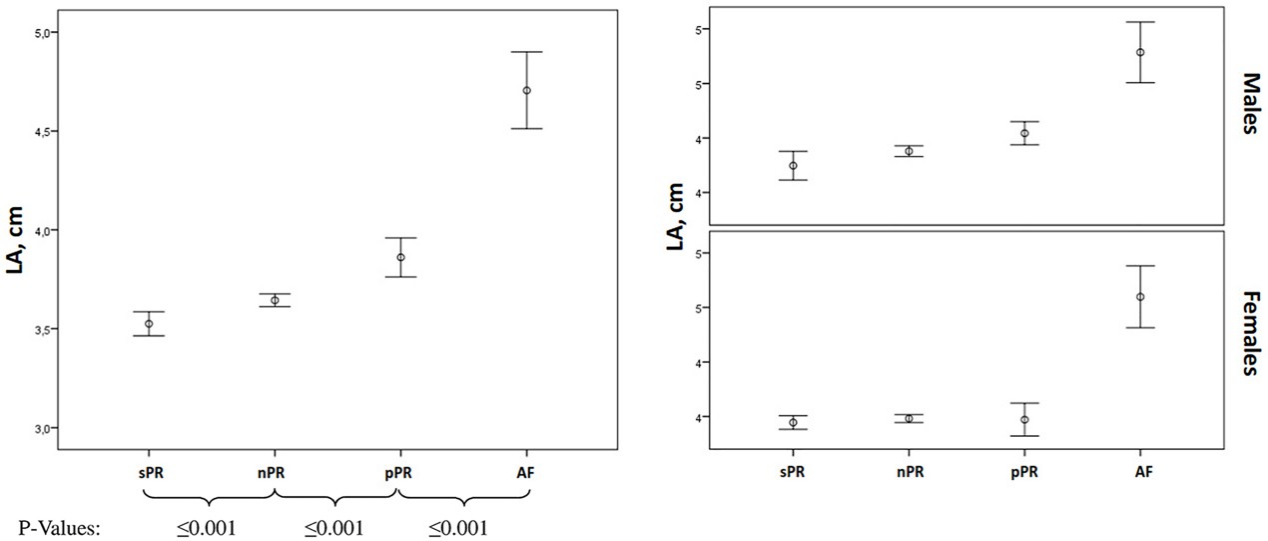
Differences between LA diameter and study groups, accordingly to PR interval length or AF in total population and gender.

LAD was significantly different between all groups (p<0.001). Comparing individuals with nPR and pPR, LAD was significantly higher in individuals with PR interval prolongation (median 37 (34–40) vs 39 (35–42) mm, p<0.001) (Table 1, Fig 1). These results became even more obvious for LAD ≥40 mm: 12.6% in sPR, 23.2% in nPR, 40.6% in pPR and 81.3% in AF; p<0.001. Similar results were observed when men and women were analyzed separately (Fig 1).

#### Troponin T

**Fig 2:**
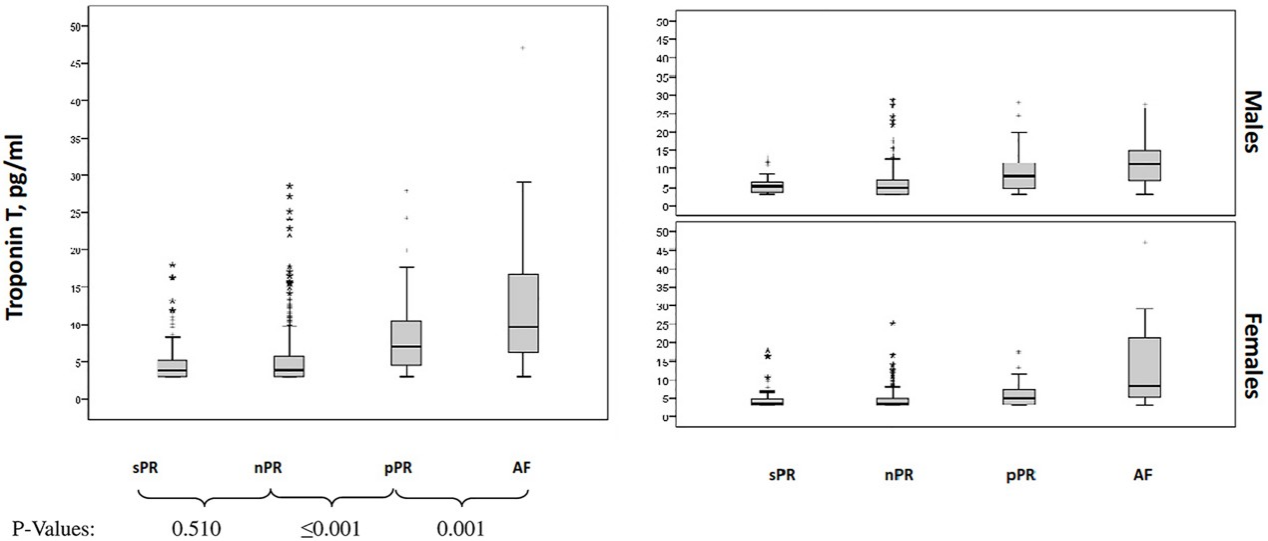
Differences between Troponin T levels and study groups accordingly to PR interval length or AF in total population and gender.

We found significant differences in TropT levels between all groups (p<0.001, Table 1). The TropT levels in the group with pPR were higher than in nPR (7.0 vs 3.9 pg/ml, p<0.001), but lower than in the AF group (Fig 2). Similar results were observed after men and women were analyzed separately.

#### NT-proBNP

**Fig 3:**
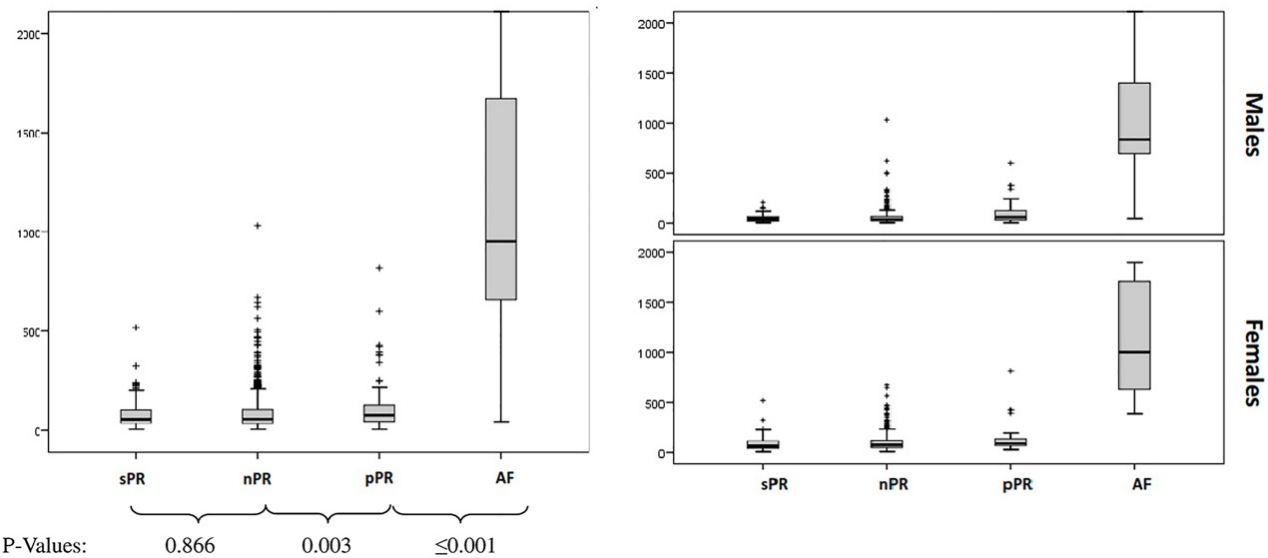
Differences between NT-proBNP levels and study groups accordingly to PR interval length or AF in total population and gender.

There were significant differences in NT-proBNP levels between all groups (p<0.001, Table 1). The highest NT-proBNP levels were observed in AF group (median 951 (IQR 640–1679) pg/ml). Individuals with sPR and nPR interval had the lowest NT-proBNP levels (median 56 (IQR 35–106) vs 58 (IQR 34–105) pg/ml). The NT-proBNP levels in the group with pPR were significantly higher than in nPR group (median 77 (IQR 45–126) vs 58 (IQR 34–105) pg/ml, p<0.001) but lower than in the group with AF (Fig 3).

#### Renal function

**Fig 4:**
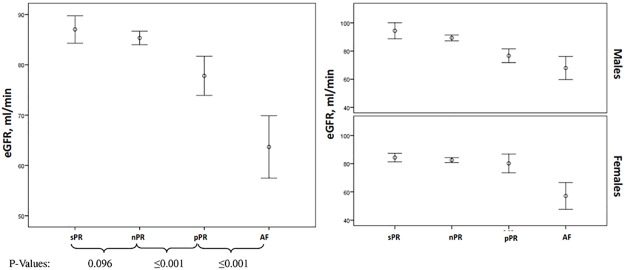
Differences between eGFR levels and study groups accordingly to PR interval length or AF and gender.

We found significant differences in eGFR and creatinine levels between all groups (p<0.001, Table 1). While individuals with sPR and nPR intervals had the highest eGFR levels (median 85 (IQR 70–100) and 82 (IQR 70–97) ml/min/1.73m^2^, respectively), we observed significant impairment in renal function in the groups with pPR interval and AF (mean 75 (IQR 61–90) and 61 (IQR 51–75) ml/min/1.73m^2^, p<0.001). These findings were observed in men, but not in women (Fig 4).

#### Other parameters

There were significant differences between groups regarding hypertension, BMI and diabetes between all groups (Table 1). Although there was a significant difference in EF between the whole cohort (p<0.001), the difference between nPR and pPR did not reach significance (p = 0.052). The analyses for heart rate >100 bpm were statistically not possible because of very low number of individuals with tachycardic profile and with sinus rhythm. As expected, individuals with AF had more frequently a heart rate >100 bpm (Table 1).

#### Logistic regression analyses

Multivariate logistic regression analyses were performed to assess whether PR interval prolongation and AF are associated with similar clinical and demographical characteristics. Three models had been chosen (Tables 2–4). Model 1 (participants with normal PR, n = 1.057 versus with PR interval prolongation, n = 138) identified following statistically significant characteristics: age (OR 1.083 per year, p<0.001), male gender (OR 2.468, p = 0.001), EF (OR 0.957, p = 0.013) and troponin>10 pg/ml (OR 2.429, p = 0.002, Table 2, Model A). Interestingly, after inclusion of LAD>40 mm, GFR<60 ml/min/1.73m^2^ and NT-proBNP>125 pg/ml as clinically important variables associated with AF, only LAD (OR 1.437, p = 0.102) and GFR (OR 1.603, p = 0.079) demonstrated strong associations, although these were not significant (Table 2, Model B).

**Table 2:** Logistic regression models—PR prolongation (n = 138) vs normal PR interval (n = 1.057), adjusted for age and gender.

Model A			Model B		
Characteristics	OR (95% CI)	*p*-value	Characteristics	OR (95% CI)	*p*-value
Age, years	1.083 (1.060–1.107)	<0.001*	Age, years	1.082 (1.057–1.107)	<0.001*
Males	2.468 (1.625–3.747)	0.001*	Males	2.211 (1.403–3.483)	0.001
EF, %	0.957 (0.924–0.991)	0.013	EF, %	0.956 (0.923–0.990)	<0.012
Troponin>10 pg/ml	2.429 (1.3824–4.266)	0.002*	Troponin>10 pg/ml	2.294 (1.287–4.089)	0.005*
			LAD>40 mm	1.437 (0.930–2.118)	0.102
			eGFR<60ml/min/1.73m^2^	1.603 (0.947–2.715)	0.079
			NT-proBNP>125 pg/ml	0.797 (0.947–2.715)	0.380

**Abbreviations:** as in Table 1; OR—odds ratio, CI—confidence interval

* significant after Bonferroni correction

**Table 3:** Logistic regression models—AF (n = 48) versus normal PR interval (n = 1.057), adjusted for age and gender.

Model A			Model B		
Characteristics	OR (95% CI)	*p*-value	Characteristics	OR (95% CI)	*p*-value
Age, years	1.104 (1.033–1.181)	0.004*	Age, years	1.104 (1.031–1.182)	0.005*
EF, %	0.824 (0.758–0.896)	<0.001*	EF, %	0.830 (0.763–0.904)	<0.001*
LA>40 mm	8.651 (3.047–24.560)	<0.001*	LA>40 mm	7.977 (2.770–22.974)	<0.001*
Troponin T>10 pg/ml	3.589 (1.282–10.041)	0.015*	Troponin T>10 pg/ml	3.042 (1.042–8.878)	0.042
eGFR<60ml/min/1.73m^2^	3.786 (1.338–10.708)	0.012*	eGFR<60ml/min/1.73m^2^	3.980 (1.404–11.283)	0.009*
NT-proBNP>125 pg/ml	42.971 (5.437–339.625)	<0.001*	NT-proBNP>125 pg/ml	49.414 (6.106–399.916)	<0.001*
			Males	1,701 (0.630–4.592)	0.294

**Abbreviations:** as in Tables 1 and 3; OR—odds ratio, CI—confidence interval

* significant after Bonferroni correction

**Table 4:** Logistic regression model–PR prolongation (n = 138) vs AF (n = 48).

Model A*		
Characteristics	OR (95% CI)	*p*-value
Age, years	1.1 (1.0–1.1)	0.588
Males	1.3 (0.5–3.2)	0.883
NT-proBNP in pg/ml*	1.011 (1.007; 1,016)	<0.001

*Using Collet method with the most stable variables.

Model 2 (normal PR, n = 1.057 versus AF, n = 48) identified the following statistically significant characteristics: age (OR 1.104 per year, p = 0.004), EF (OR 0.824, p<0.001), LAD>40 mm (OR 8.651, p<0.001), Troponin>10 pg/ml (OR 3.589, p = 0.015), GFR<60ml/min/1.73m^2^ (OR 3.786, p = 0.012) and NT-proBNP>125 pg/ml (OR 42.971, p<0.001, Table 3, Model A). After adjustment for gender, all variables remained significant (Table 3, Model B).

In Model 3 (comparison between PR interval prolongation and AF), the only parameter that was found to be stable within both groups was NT-proBNP (p<0.001) (Table 4).

## Discussion

### Main findings

In this cross-sectional analysis, we demonstrate significant associations between left atrial diameter, renal function and biomarker of cardiac damage–high-sensitive Troponin T–with PR interval prolongation. Furthermore, our analyses demonstrate that PR interval prolongation and AF share similar characteristics. Only NT-proBNP levels were significantly higher in AF than in PR interval prolongation.

### PR interval prolongation as preliminary stage for AF

AF is the most common sustained arrhythmia and it is expected that every fourth adult will develop AF throughout life [4]. Because of its association with an increased risk of dementia, heart failure, and thromboembolism, the treatment of AF complications as well as increased hospitalization and mortality lead to higher treatment costs [13].

The aim of AF prevention is to predict and timely recognize important factors associated with higher risk for AF development and perpetuation. One of these tools might be an ECG–an easily available, cost effective and informative diagnostic tool in clinical routine. Different studies analyzed the role of PR interval prolongation on AF incidence [1]. The electrocardiographic PR interval reproduces the atrial and atrioventricular conduction. So far, PR prolongation without structural heart disease or additional conduction disturbances has been considered as a benign occurrence [2]. However, recent studies demonstrated an association between PR prolongation and the underlying atrial remodelling processes [14] leading to increased AF incidence [2,3]. A significant correlation between PR interval prolongation and AF recurrence after radiofrequency ablation was also shown [15]. Furthermore, Schumacher et al recently demostrated that PR interval prolongation is associated with electro-anatomical substrate in AF patients, assuming that PR interval could be used as a marker for atrial remodelling before catheter ablation [16].

### Association with clinical, imaging and blood biomarkers

In the current analysis we found several important clinical and blood biomarkers associated with PR interval prolongation and AF. LA diameter is significantly higher in AF patients and indicates structural remodeling. It is associated with AF progression and recurrences after catheter ablation [17]. In our study we found that LA diameter–as an easy obtainable imaging biomarker–was larger in PR interval prolongation than in normal PR, but smaller than in individuals with AF.

Another important factor associated with AF and adverse outcomes after different therapeutic strategies is renal function. The role of cardio-renal axis in AF patients had been analyzed in several studies. A bidirectional relationship between AF and kidney dysfunction had been described [18]. This suggests mutual molecular pathways in both AF and renal dysfunction. While individuals with chronic kidney disease are more likely to develop AF, thromboembolic events and bleeding [19,20]. patients with renal impairment are at higher risk for cardio- and cerebrovascular complications. Furthermore, there is a correlation between LA enlargement as a sign for structural remodeling reflecting a chronic exposure to hemodynamic overload due to renal disease and AF in a synergetic way [21]. Moreover, Majima et al. demonstrated an association between PR interval and eGFR decline in healthy subjects [22] that is in accordance with our results. In current study we found that renal function in individuals with PR prolongation was worse than in individuals with normal PR interval, but significantly better than in chronic AF. Furthermore, in the multivariate model, the renal dysfunction was the one of the factors strongly associated with AF. Of note, renal dysfunction was associated also with PR interval prolongation, however this result did not reach significance. We suppose that by weaker association than in AF, larger number of individuals with PR interval prolongation would be needed to reach significance level. Nevertheless, these findings support our hypothesis that PR interval prolongation might be considered as preliminary stage for AF.

As a marker of cardiomyocytes damage, TropT plays an important role in an ischemic heart disease. However, its impact is relevant also in other cardio- and cerebrovascular comorbidities, such as hypertension, heart failure, stroke, or renal dysfunction. Recently, it had been shown that in chronic heart failure increased TropT predicts all-cause and cardiovascular mortality [23]. Therefore, it is assumed that TropT release is a consequence of myocardial ischemia of any cause or cardiomyocyte damage caused by inflammatory infiltration and myocardial apoptosis [23]. Recently, it has been demonstrated that increased TropT levels are associated with AF incidence [24]. Furthermore, the importance of TropT has been analyzed in large AF cohorts and implemented into the ABC scores for the prediction of thromboembolic and bleeding complications as well as mortality in AF patients [25,26]. In our study, there were significant differences between Troponin levels in individuals with normal and prolonged PR interval and AF [8]. Furthermore, Troponin levels in individuals with PR interval prolongation and AF were significantly higher than in individuals with short and normal PR intervals. Also, we found that PR interval prolongation and AF share similar characteristics. In multivariate model age, worse EF and larger LA as well as Troponin levels were the common factors in individuals with PR interval prolongation and AF.

### Heart failure and NT-proBNP

The prevalence of AF in patients with heart failure (HF) ranges from 13% to 27% [27]. In the Framingham Heart Study, HF was associated with AF risk in both genders, however, the association was significantly higher in women [28]. The strong association between HF and AF has been attributed to shared mechanisms leading to neurohormonal and proinflammatory activation, which induces myocardial inflammation and fibrosis. The atrial substrate with HF is characterized by atrial fibrosis and abnormalities in Ca^2+^ handling. These changes are distinct with electrophysiological abnormalities in AF-induced atrial remodeling [29]. Of note, recent studies demonstrated that PR interval prolongation is common in patients with HF with both reduced and preserved EF and is associated with worse survival although not an independent predictor of outcome [30].

NT-proBNP plays the most important role in HF patients. However, this biomarker is also important in prediction of clinical outcomes in patients with AF [31]. Using a multi-biomarker approach, NT-proBNP was the strongest predictor of incident AF and improved the predictive ability when added to traditional risk factors [32]. In other studies, NT-proBNP was an important part of a novel biomarker-based score–ABC (age, biomarkers, clinical history)–which demonstrated significant association predicting stroke, bleeding and, finally, death [25,26]. Also, patients with AF develop often clinical HF symptoms or even EF decrease (e.g. tachycardiomyopathy). Furthermore, there is an association between increased AF incidence in HF and increased HF (symptoms) in AF patients. This is a possible explanation regarding the role of NT-proBNP levels predicting mortality in AF patients [26].

In our study, the only significant difference between AF and PR interval prolongation had been found in NT-proBNP levels. This might be explained by higher heart rates in AF patients leading to HF symptoms–and consequently higher NT-proBNP levels. Also, relatively low NT-proBNP levels in individuals with PR interval prolongation indicates rather ‘stable’ cardiac homeostasis without necessity to release biomarkers of cardiac stress. Largely, it could be explained by the normofrequent heart rhythm. Whether the NT-proBNP levels differ in patients with long standing persistent (chronic) normofrequent AF and paroxysmal AF with tachyarrhythmic phase of arrhythmia, could not be proved in current epidemiological setting and should be addressed in clinical studies.

### Strengths and limitations

Out of 10.000 individuals recruited in the LIFE-Adult-Study, at the time of analysis, ECG data (readings) were available in 4.621 individuals, while echocardiographic data (readings) were available in 1.750 individuals. This is the main limitation of current study. Nevertheless, despite cross-sectional *interim* analysis, up to date this is the largest study addressing this issue. In the present study, we could not definitely confirm that PR prolongation is a predictor of AF. The cross-sectional character of our analysis is only a hypothesis generating step forwards this suggestion. In case this hypothesis should be confirmed by longitudinal data, it will be an important step identifying individuals at higher risk for AF using simple tools as biomarker and ECG.

## Conclusions

Individuals with PR interval prolongation and AF showed similarities in echocardiographic parameters, renal function and blood biomarker levels. Longitudinal studies are need to prove whether the PR interval prolongation might be considered as preliminary stage for AF. This could be helpful identifying individuals at higher risk for AF using biomarker and ECG assessment.
